# Diabetic nephropathy among Mexican Americans 

**DOI:** 10.5414/CN107487

**Published:** 2012-02-20

**Authors:** Subrata Debnath, Farook Thameem, Tahira Alves, Jacqueline Nolen, Hania Al-Shahrouri, Shweta Bansal, Hanna E. Abboud, Paolo Fanti

**Affiliations:** 1 Division of Nephrology, University of Texas Health Science Center at San Antonio and; 2 Renal Section, Audie L. Murphy VA Hospital, San Antonio, TX, USA

**Keywords:** diabetes, nephropathy, Mexican American, epidemiology

## Abstract

The incidence of diabetic nephropathy (DN) is growing rapidly worldwide as a consequence of the rising prevalence of Type 2 diabetes mellitus (T2DM). Among U.S. ethnic groups, Mexican Americans have a disproportionately high incidence and prevalence of DN and associated end-stage renal disease (ESRD). In communities bordering Mexico, as many as 90% of Mexican American patients with ESRD also suffer from T2DM compared to only 50% of non-Hispanic Whites (NHW). Both socio-economic factors and genetic predisposition appear to have a strong influence on this association. In addition, certain pathogenetic and clinical features of T2DM and DN are different in Mexican Americans compared to NHW, raising questions as to whether the diagnostic and treatment strategies that are standard practice in the NHW patient population may not be applicable in Mexican Americans. This article reviews the epidemiology of DN in Mexican Americans, describes the pathophysiology and associated risk factors, and identifies gaps in our knowledge and understanding that needs to be addressed by future investigations.

## Introduction 

Mexican Americans are a unique racial mix of American Indians and Europeans, especially Spaniards [1]; they represent 2/3 of Hispanic Americans and are the largest and fastest growing ethnic minority in the United States [2, 3]. Despite objective difficulties in discriminating between Mexican Americans and other Hispanic American subgroups based on current official definitions [4, 5] and medical literature, the available data suggest that Mexican Americans are at a greater risk than other subgroups for developing T2DM and its micro- and macrovascular complications including nephropathy (T2DN), retinopathy, neuropathy and cardiovascular disease [6]. Therefore, it is not surprising that the health of Mexican Americans is of increasing concern to U.S. health-care providers, researchers, and policy makers. This paper reviews the current knowledge and understanding of T2DN in Mexican Americans and attempts to identify the major knowledge gaps and disease management deficiencies specific to the Mexican American population. The hope is that this information will serve as a foundation upon which future research and clinical initiatives can be based. 

## Methods 

We searched the electronic databases PubMed, EMBASE, SCOPUS, Web of Science and CINAHL for original articles based on the following inclusion criteria: publication in the English literature after 1970, full report of original cross-sectional, prospective, or observational human studies, evaluation of T2DM and/or T2DN, and analysis of incidence, prevalence, progression and/or complications. We identified articles of interest based on the following key words: adult-onset diabetes mellitus, T2DM, maturity onset diabetes mellitus, non-insulin dependent diabetes mellitus (NIDDM), hyperglycemia, metabolic syndrome, diabetic nephropathy, chronic kidney disease, diabetic kidney disease, uremia, renal failure, end-stage renal disease (ESRD), hemodialysis, minorities, health disparities, Hispanics, Mexican Americans, Latinos, obesity, central obesity, body mass index (BMI), socioeconomic status, acculturation, genetics, migration, diet, nutrition, lifestyle risk factors, environmental risk factors, incidence, prevalence, prevention, etiology, complications, progression, treatment, intervention, management, alternative medicine. In addition, we screened relevant citations from review articles and trials to identify articles that were not found through the database searches. We methodically identified studies that focused on Mexican Americans or Hispanics/Latinos and that targeted geographic areas where Mexican Americans are clustered including West (California) and Southwest (Texas). Studies on NHWs were also analyzed for comparison. One objective limitation of this literature search and analysis originates from the lack of consensus about the definition of Mexican Americans in health-related research. For example, the Hispanic Health and Nutrition Examination Survey (HHANES) [5] defined Hispanic subgroups based on self-reported national origin or ancestry, while Hazuda et al. [6] emphasized the importance of parental surnames as indication of Mexican-American ethnicity. With few exceptions, e.g. the San Antonio Family Diabetes/Gallbladder Study (SAFDGS) and the San Antonio Family Heart Study (SAFHS), most of the reviewed studies used self-reported ethnicity to identify Mexican Americans. 

## T2DN among Mexican Americans 

Hispanic Americans are the ethnicity with the highest estimated lifetime risk of diabetes in the US [7]. Further, surveys conducted in the 1990’s demonstrated that both the incidence and prevalence of T2DM were at least 2-fold higher in the Hispanic subgroup of Mexican Americans than in NHW and were substantially higher in Mexican Americans than in any other Hispanic subgroup except for Puerto Ricans [8, 9, 10, 11, 12, 13, 14]. Not surprisingly, a similar incidence and prevalence of T2DN was also observed in this population [6, 15, 16, 17]. For example, in the San Antonio Heart Study (SAHS), micro- and macroalbuminuria were observed in 26% and 11%, respectively, of Mexican Americans compared to only 9% and 5% of NHWs [16]. This heightened susceptibility of Mexican Americans to T2DM and T2DN is the object of ongoing study and debate and is the focus of this paper. 

The American Indian genetic pool is believed to contribute substantially to the high rates of T2DM and T2DN among Mexican Americans. Indeed, T2DM and T2DN occur with extremely high frequency among American Indians, as extensively documented among the Pima Indians who presumably have close to 100% native American genes [18]. The rate of T2DM in Mexican Americans has been suggested to parallel the percent of gene pool derived from the American Indian population [19]. A prospective study of Pima Indians with T2DM also demonstrated an exceptionally high percent of patients (37%) who progressed from micro- to macroalbuminuria over a 4-year period [20]. In the same population, a 20-year history of T2DM was associated with a 50% cumulative incidence of macroalbuminuria [21]. The latter, in turn, was associated with a 42-fold higher progression toward ESRD, confirming the role of albuminuria as an ominous prognostic marker of end organ failure in this ethnic group [21]. 

Interestingly, more recent surveys contradict the above observations by reporting a lower prevalence of chronic kidney disease (CKD) in Mexican Americans than in NHWs [11, 22, 23, 24, 25]. The authors of these latter studies offer several possible explanations for this incongruence including race- and ethnicity-based differences in the 1) accuracy of the equations used to estimate glomerular filtration rate (eGFR), 2) diagnostic tests for microalbuminuria, 3) progression rate of CKD, 4) death rate prior to reaching ESRD, and 5) access to health care [23, 25, 26]. To our knowledge, the only study that has attempted to further clarify this issue was a retrospective observational study that compared random samples of Mexican American, African American and NHW patients with ESRD due to Type II DM (ESRD-DM). In this study, the rate of progression of renal disease was faster in Mexican Americans than in the other ethnic groups and was shown to be independent of blood pressure and glycemic control [27]. Based on these findings the authors concluded that other yet to be identified risk factors for ESRD-DM beyond the traditional risk factors of blood pressure and glycemic control are present in the Mexican American population. If confirmed to be true this could have significant implications for nephrologists and other health care professionals who care for the Mexican American population since it would set Mexican Americans apart from other patient populations in whom glycemic and blood pressure control are important determinants of the incidence and rate of progression of T2DN [28, 29] . Finally, further complicating our understanding of the relationship between T2DM, T2DN, and ESRD-DM in the Mexican American population is the recently emphasized inaccuracy of albuminuria as a biomarker of CKD; in fact, a relatively large fraction of T2DM patients, including Mexican Americans, present with normoalbuminuria despite an estimated glomerular filtration rate (eGFR) < 60 ml/min/1.73 m^2^ [30, 31, 32, 33]. This latter observation implicates the existence of other pathogenetic expressions of T2DN which possibly may be dependent in part on genetic and lifestyle factors. 

## ESRD among Hispanic Americans and Mexican Americans with T2DM 

National statistics regarding ESRD in Hispanic Americans became available after 1994 when Medicare and Medicaid introduced an ethnicity query on the 2,728 ESRD Registration form. Based on data from the United States Renal Data System (USRDS), the prevalence of ESRD-DM has been 2-fold higher in Hispanics than in non-Hispanics since 2001 [34, 35]. During this time period, the incidence rate has been relatively stable with 311 – 326 new cases/million Hispanics, while the prevalence rate has increased by 13%, from 1,141 to 1,306 cases/million, probably as a result of improved survival with renal replacement therapy [13, 14]. 

Unfortunately, prospective data of comparable quality are not available in the Mexican American subgroup of Hispanic Americans. However, an epidemiological survey conducted in Southern-Central Texas in the 1980’s found a 4-fold to greater than 9-fold higher incidence of ESRD in Hispanics – almost all Mexican Americans – than in NHW [15]. In 1995, a combined survey of the San Antonio and Dallas areas confirmed that an impressive 93% of Mexican Americans with ESRD also suffered from T2DM [36] compared to a 40% prevalence of T2DM in the overall ESRD patient population in the U.S. Between 1996 and 2006, the Mexican Americans experienced a faster incident growth of ESRD compared to African Americans and Native Indians [35, 37]. Another interesting observation is that the distribution of ESRD-DM by U.S. state correlates relatively well with that of Mexican Americans. For example, in 2007 the incidence of ESRD-DM was highest (183 – 225 cases/million population) in California, Arizona, West Virginia, New Mexico, Hawaii, and Texas, i.e. mostly states with large Mexican American populations, while it was lowest (83 – 110 cases/million population) in Wyoming, Vermont, Montana, New Hampshire, Maine, and Oregon, i.e. states with low Mexican American population [38]. Although these data are obviously biased by lack of control for recent immigration and naturalization trends in the U.S. they suggest a strong association between ethnicity and the incidence of ESRD-DM in the U.S. [34, 35] and they allow us to predict a significant impact of Mexican Americans on the future incidence of ESRD in the U.S. 

In addition, Hispanics with ESRD have also been estimated to have a 20 – 30% lower mortality risk than their NHW counterparts [39, 40]. The survival advantage of Hispanics compared to NHWs has been observed repeatedly despite the higher prevalence of T2DM in this patient population, a phenomenon often referred to as the “Hispanic Paradox” [39, 41, 42]. Further, in a recent subgroup analysis, Mexican and Cuban Americans with ESRD were also shown to have better survival compared to Puerto Ricans and non-Hispanics [42]. The survival advantages in both Hispanics and the Hispanic subgroup of Mexican Americans have been attributed to age, BMI, serum albumin, blood hemoglobin concentrations, and hemodialysis adequacy [39, 41, 42]. For example, high BMI in Hispanic ESRD patients is associated with “reverse epidemiology” which confers a benefit toward survival [43, 44]. 

## Genetic predisposition to T2DN among Mexican Americans with T2DM 

Epidemiological studies strongly implicate genetics as a major contributor to the development, progression and heritability of T2DN as of T2DM [45]. Genetic studies in Mexican Americans support this as demonstrated by the finding of familial clustering of T2DN and related phenotypes in this population. However, despite mounting appreciation for the influence of genetic variation on the risk for development of chronic diseases, the specific gene(s) involved in the susceptibility to T2DN and related phenotypes remains elusive [46, 47]. T2DN susceptibility genes have been investigated in Mexican Americans as part of several recent large studies, including the Family Investigation of Nephropathy and Diabetes (FIND) [48], the SAFDGS [49], the SAFHS [50], and the National Health and Nutrition Examination Survey (NHANES) [51]. Linkage and biological candidate gene analyses, mapping by admixture linkage disequilibrium (MALD) and genome-wide association studies are some of the strategies that have been used in these studies to identify susceptibility loci and genes for T2DN and related phenotypes. 

In Mexican Americans recruited for FIND, genome-wide linkage analysis reported an association between mutations of certain chromosomal regions with the presence of T2DN (9q33), albuminuria (9q31) and GFR (1q43, 2p13, 7q36.1, 8q21.3, 18q23) [52, 53]. In SAFDGS, the same analytical methodology suggested an association between albuminuria and variation in the *GABRB3*-flanking region on chromosome 15q12 [54] and between GFR and the *D2S427*-flanking region on chromosome 2q36. However, within the *GABRB3*-flanking region genetic variations of the positional candidate genes tight-junction protein-1 (*TJP1*) and gremlin-1 (*GREM1*) failed to show evidence of an association with albuminuria [55, 140]. 

A quantitative trait linkage scan of subjects recruited for SAFHS showed the strongest association of albuminuria, serum creatinine and GFR with variations in chromosome regions 20q12 and 9q21, respectively [57, 58]. In the same cohort, GFR and serum creatinine also displayed somewhat weaker associations with the 2p25 region [58]. 

In addition, genes involved in the regulation of blood pressure, endothelial biology and redox functions have also been investigated in Mexican Americans as possible candidate genes for susceptibility to T2DN and related phenotypes. Unfortunately, genetic variations that have previously yielded promising results including those of endothelial nitric oxide synthase (eNOS), paraoxonase 2 (PON2) and components of the renin-angiotensin system failed to demonstrate any strong associations with albuminuria or GFR in the SAFDGS and SAFHS cohorts [56]. Chu et al. [59] also did not find any association between the e2/e4 alleles of *APOE* and GFR in 1,656 Mexican Americans from NHANES III. Use of MALD in Mexican Americans in the FIND study led to the novel observation of an association with the genetic variants of hemicentin 1 but failed to confirm the previously reported association of T2DN with carnosine dipeptidase 1 (*CNDP1*) and engulfment and cell motility 1 (*ELMO1*) [60]. 

In summary, the identification of several candidate gene loci has not yet resulted in the discovery of major susceptibility gene for T2DN in Mexican Americans. Still, recent identification by linkage and candidate gene analysis of calpain 10 (*CAPN10*) and transcription factor 7-like 2 (*TCF7L2*) as susceptibility genes for T2DM in Mexican Americans offers hope for the future discovery of T2DN-specific genetic traits in this same population [61, 62, 63, 64, 65, 66]. 

Collectively, these analytical tools may assist in accelerating the identification of gene variations that may contribute to the development and progression of T2DN in Mexican Americans and possibly other ethnic populations. In addition, ongoing discovery of proteins and other signaling pathways that are functionally relevant to the pathophysiology of T2DN will provide opportunities for further testing of new genetic variations. 

## Other risk factors, prevention and treatment of T2DN among Mexican Americans with T2DM 

Current standard practice is to manage T2DM and T2DN in Mexican Americans using the same basic principles of risk, prevention, and treatment that are applied to the general population. However, as discussed in this section, evidence-based data from prospective clinical trials underscore the need for more studies that specifically target ethno-specific approaches to the care of T2DM and T2DN within the Mexican American population. 

### Hypertension

The NHANES have consistently reported lower prevalence of hypertension in the general and T2DM Mexican American population than in their NHW and African American counterparts [17, 67, 68, 69, 70, 71, 72]. This phenotypic trait can possibly be explained by the previously mentioned contribution of American Indians to the Mexican Americans genetic pool and by the observation that American Indians, specifically the Pima Indians, do not experience the same positive correlation of blood pressure with insulin resistance and T2DM that is present in NHWs [73, 74]. Other plausible explanations for the lower incidence of hypertension in Mexican Americans with T2DM may be due to differences in disease diagnosis, awareness, treatments, and adherence to therapy among Mexican Americans compared to African Americans or NHWs [17, 70, 72, 75, 76, 77]. Interestingly, recent data from the Hispanic-Chronic Renal Insufficiency Cohort study seem at odds with the above evidence in the general diabetic population, since they suggest that once CKD is established, the prevalence of hypertension becomes higher in Mexican Americans with CKD than in NHW with CKD, even though this study did not stratify for diabetes status [78]. With regard to treatments, it is interesting that antihypertensive medications particularly well-suited to protect against the development and progression of T2DN, including the angiotensin receptor blockers (ARBs) and angiotensin converting enzyme inhibitors (ACE-I) [79, 80, 81], are not as commonly prescribed in Mexican Americans as in other ethnic groups with diabetes [82, 83, 84]. In addition, the efficacy of these classes of medications in Mexican Americans compared to other ethnicities has recently been questioned. In a post-hoc analysis of an international study, the ARB losartan was shown to provide weaker protection from progression to ESRD in Hispanics compared to NHWs, African Americans and Asians although the effect on proteinuria was comparable among groups [85]. Analysis by the Agency for Healthcare Research and Quality [86] highlights the scarcity of comparative information on benefits and harms of ACE-I and ARBs in minority populations including Mexican Americans. More research is needed that aims to determine the benefits of these agents among often understudied minority populations. 

### Hyperglycemia

Several observational and interventional studies across ethnic and racial groups, including the Diabetes Control and Complications Trial (DCCT), the United Kingdom Prospective Diabetes Study (UKPDS), and the Steno-2 study, indicate that optimal glycemic control, independent of blood pressure control, prevents or reverses the early manifestations of nephropathy in patients with T2DM [87, 88, 89, 90, 91, 92]. Unfortunately, cross-sectional and prospective studies of both incidental and established T2DM have consistently shown that Mexican Americans experience inferior quality of care and glycemic control compared to other ethnic groups [17, 71, 93, 94, 95, 96, 97, 98, 99]. For example, between 1988 and 2002, HbA1c was found to be greater than 9.5% in 22% of Mexican Americans compared to 16% of NHWs with T2DM. In addition, there was no evidence of improvement in this disparity during long-term follow-up [100, 101]. Furthermore, it was observed that only 28% of Mexican Americans self-monitor blood glucose as compared to 44% of NHWs and 36% of African Americans, a practice associated with a healthier lifestyle in patients with T2DM, particularly those not using insulin [96]. 

### Dyslipidemia

Elevated serum cholesterol has been shown to predict the development of albuminuria in patients with T2DM [102], although it is unknown whether this finding holds true in Mexican Americans. In addition, the prevalence of dyslipidemia in the Hispanic population is uncertain. In one report, the presence of hypercholesterolemia and hypertriglyceridemia were 36% and 42% less common, respectively, in Hispanics compared to NHWs with T2DM[103]. More recently, a multi-ethnic study of atherosclerosis reported comparable prevalence of dyslipidemia in Hispanics and NHWs, although the Hispanic subjects were less likely to be appropriately treated [104]. Compared to NHWs, it has also been demonstrated that Mexican Americans are less aware of suffering from dyslipidemia (55% vs. 33%) and are less likely to be treated (30% vs. 14%) [97]. Similar observations have also been made when comparing Mexican Americans and NHWs regarding T2DM. In addition, Mexican Americans with and without T2DM are approximately 30% less likely than NHWs to be diagnosed and treated with diet or medications for their dyslipidemia [17]. Unfortunately, there are no data currently available describing the prevalence and pathogenetic significance of dyslipidemia in Mexican Americans with T2DN. 

### Obesity

A causal role of obesity in the pathogenesis of T2DN has been hypothesized [97] although the available clinical studies are inconsistent. A European longitudinal study found no independent association between obesity and T2DN [105]. However, more recently the Look AHEAD study reported a positive correlation between abdominal obesity but not total body fat and albuminuria in T2DM subjects [106]. Furthermore, a meta-analysis has concluded that a reduction in body weight leads to lower proteinuria and microalbuminuria in T2DM, although it is not known if this translates into a reduction in the incidence of ESRD in clinical practice [107]. 

Despite a very high prevalence of obesity among Mexican Americans, we are not aware of any analysis that specifically addresses the association between obesity and T2DN in this population. It has however been shown that age, duration of diabetes, retinopathy, hypertension, and cardiovascular disease are among the most significant predictors for the development of nephropathy in obese Mexican Americans with T2DM [108]. 

### Life-style and socio-economic status

Risk factors for insulin resistance that depend on life-style and socio-economic status are highly prevalent in Mexican Americans, including accumulation of abdominal visceral fat with central obesity [109, 110], excess alcohol consumption, lack of physical activity [111], and lower education level [69]. This is relevant since insulin resistance is the major metabolic abnormality in T2DM and the prevalence of risk factors for insulin resistance in Mexican Americans may be contributing significantly to the excess T2DM in this population [112, 113, 114, 115, 116, 117]. In a recent cross-sectional analysis, physical activity correlated with both glomerular filtration rate (GFR) and proteinuria in NHWs and with GFR in Mexican Americans [118]. Additionally, erratic management of T2DM, mostly a consequence of socio-economic and access barriers, has been correlated with an increased risk of progression of kidney disease in elderly Mexican Americans [119]. 

### Alternative therapy for T2DN

Use of complementary and alternative medicine (CAM) for T2DM and T2DN is a very prevalent practice among Mexican Americans, presumably as a consequence of their strong ties to their culture, relatively low socioeconomic status, and inadequate access to conventional medical care. Small studies have shown that *nopal* (prickly pear cactus) and *aloe vera* are widely used as CAM by Mexican Americans with T2DM [120, 121, 122, 123]. Poss et al. [124] also reported that *Te Diabetil*, a combination of several herbs, is frequently used by Mexican Americans for the management of T2DM along with prescribed allopathic medicine. Unfortunately, the National Health Interview Survey (NHIS), a large cross-sectional survey on health status and use of CAM in adults with diabetes, and other studies based on this survey [125, 126, 127] did not specifically addressed the use of CAM in Mexican Americans. In addition, none of the above studies specifically addressed the use of any of these CAM remedies for the treatment of T2DN. Markell [128] suggests the potential benefits of complementary medicines for CKD, however, the etiology of CKD is not specified and therefore the relevance to T2DM is not known. In summary, the current knowledge regarding these therapies is abysmally insufficient to determine efficacy and one must remain well aware that, contrary to many consumer expectations, CAM remedies may actually be harmful. On the other hand, it is possible some of these therapies may actually provide not yet recognized modalities of treatment that are useful especially for aspects of the disease that are not adequately addressed by current conventional therapy. For example, excessive oxidative stress has been identified as a possible contributor to the pathogenesis of many forms of disease including both T2DM and CKD [129, 130, 131, 132]. Although conventional Western medicine does not currently offer interventions that directly address oxidative stress and its associated redox defects it is possible that herbal remedies, supplements, or a group of compounds referred to as nutraceuticals that contain antioxidants may correct these defects in T2DN and other forms of CKD. At present this hypothesis is untested and requires further evaluation in both the preclinical and clinical research settings. 

### Prevention of T2DN

Identification of subjects at risk for T2DM, or with established pre-diabetes, early T2DM, or microalbuminuria are self-evident important steps for prevention of both T2DM and T2DN [133, 134]. Unfortunately, despite particularly long duration of T2DM in Mexican Americans, diagnosis tends to be made late in this population [135, 136]. Culturally sensitive efforts are underway to mitigate T2DM among Latinos as described by the Lawrence Latino Diabetes Prevention Project [137] and the La Diabetes y La Unión Familiar [138], among others. We are not aware of any programs to reduce or prevent the burden of T2DN among Mexican Americans specifically [139]. We submit, however, that a renewed effort from primary and specialty health care providers to educate patients about healthy lifestyle, to diagnose in timely manner T2DM and microalbuminuria and to refer early to a nephrologist would be steps in the right direction. This approach should consider and respect Mexican American culture and values including language, religion, health beliefs, and diet, as well as the community context – extended family and support systems – and the challenges of acculturation. 

## Conclusions 

T2DN is highly prevalent among Mexican Americans. The medical impact of this disease is extensive and is of concern to health care providers and the overall U.S. health care system, especially in consideration of the rapid demographic growth of Mexican Americans in the U.S. Many modifiable and non-modifiable risk factors of T2DN have been identified or proposed in this vulnerable population. T2DM and hypertension are paramount among the modifiable biological risk factors. In addition, low formal education level, lack of health insurance and limited access to health care have been identified as modifiable socioeconomic factors that, if left unchallenged, will interfere with any attempt aimed at correcting the biological predictors. 

This review highlights some important differences in the incidence, prevalence and risk of T2DN in the Mexican American population. The available evidence suggests that unique social, clinical and pathogenetic factors bear on the incidence and prevalence of T2DN among Mexican Americans. Clinical management of T2DN in this ethnic group may therefore benefit from direct confrontation and resolution of these ethnicity-specific differences. Further, although the identification of gene variations that predispose Mexican Americans to T2DN has progressed slowly during the last 2 decades, this effort is far from complete and should continue. 

We acknowledge the need for further studies in this patient population. We anticipate that future studies will include this high-risk population so that the natural history of T2DN can be further elucidated and that scientific evidence can be obtained that will assist in the development of future recommendations for screening, prevention, and treatment. 

## Acknowledgments 

This work was supported by grants to Subrata Debnath (AHA#0525206Y), Farook Thameem (American Society of Nephrology), Paolo Fanti (NIH#AT004490 and VA#1I01CX000264), and Hanna Abboud (NIH# DK-R01-078971 and VA Merit Review). 
